# Does Stereotype Threat Affect Men in Language Domains?

**DOI:** 10.3389/fpsyg.2020.01302

**Published:** 2020-07-08

**Authors:** Kathryn Everhart Chaffee, Nigel Mantou Lou, Kimberly A. Noels

**Affiliations:** ^1^Département de Didactique, Université du Québec à Montréal, Montreal, QC, Canada; ^2^Intercultural Communication Lab, Department of Psychology, University of Alberta, Edmonton, AB, Canada

**Keywords:** stereotype threat, language learning, gender stereotypes, education, gender equity, stereotypes, language motivation, language aptitude

## Abstract

Boys and men tend to underperform in language education, and they are also underrepresented in language-related fields. Research suggests that stereotypes can affect students’ performance and sense of belonging in academic subjects and test settings via stereotype threat. For example, girls and women sometimes underperform on math tests following reminders that math is for boys. We sought to test whether stereotypes that women have better language skills than men would affect men. In a series of four experiments (*N* = 542), we tested the effect of explicit stereotype threats on men’s performance in language-related tasks, and their sense of belonging to language-related domains. We found little evidence for stereotype threat effects on men in language. Bayesian analysis suggested that the null hypothesis was consistently more likely than the alternative, and mini-meta analyses showed effect sizes near zero. Future research should explore other explanations for gender gaps in language.

## Introduction

Previous research has established that men and boys differ from women and girls in their language motivation, participation, and test scores (e.g., Meece et al., 2006; Stoet and Geary, 2013; National Center for Education Statistics [NCES], 2014; Dewaele et al., 2016). This difference may parallel some of the issues related to women’s underparticipation and underperformance compared to men in STEM (science, technology, engineering, and mathematics) disciplines. Many studies have implicated gender stereotypes of ability and gendered images of scientists in STEM-related gender gaps (e.g., Cejka and Eagly, 1999; Clark et al., 2016; Cheryan et al., 2017). We examined whether these same stereotypes affect men: according to stereotypes, math and science are for boys, but on the other side of the same coin, verbal skills and foreign languages are for girls. Evidence of the complementary stereotype that language is feminine is widespread and well-established in both language generally (Plante et al., 2009, 2019; Steffens and Jelenec, 2011; Passolunghi et al., 2014) and foreign language domains specifically (Williams et al., 2002; Chaffee et al., 2020). Indeed, Plante et al. (2009) found that stereotypes that language is for women/girls were more robust and consistent among Canadian boys and girls than stereotypes that math is for men/boys. Research has demonstrated that both students and their teachers believe that girls are better at language in general than boys and that boys internalize these stereotypes throughout their school years (Hartley and Sutton, 2013; Retelsdorf et al., 2014).

Because men, like women, are subject to gendered stereotypes about their aptitude, we examine the proposition that some of the same issues and stereotypes that deter women from entering STEM disciplines may also limit the occupational interests and opportunities of men and boys by deterring them from enrolling and engaging in language classes. Stereotypes about language ability and languages as a female domain are likely to affect students in a number of ways. One of the most well-studied mechanisms for the influence of stereotypes on women in STEM is stereotype threat. Studying whether stereotype threat impacts men in language may provide insights for understanding men’s underperformance and underrepresentation in language.

### Stereotype Threat

Stereotype threat refers to the phenomenon that individuals who are the target of a negative stereotype feel pressured not to confirm that stereotype, and this pressure leads to poor performance by distracting from the task (e.g., Aronson et al., 1999; but see the Discussion for a more critical picture). It is important to note that for stereotype threat effects to occur, it is not necessary for the targeted individual to believe the stereotype is true; awareness that others in society may hold this stereotype is what leads to stereotype threat (Spencer et al., 1999; Leyens et al., 2000; Pennington et al., 2016). Because stereotype threat increases the cognitive load of a task by forcing the person to attend to both the task and the pressure of the stereotype, stereotype threat is thought to affect performance particularly on difficult tasks and tests (Nguyen and Ryan, 2008, p. 1324), although this may be true especially among individuals high in domain identification (Keller, 2007a). In a classic study of stereotype threat, female university students performed more poorly on a challenging math test when reminded of the stereotype that males are better than females at math compared to when the stereotype was not salient (Spencer et al., 1999). Further studies have suggested that the effects of stereotype threat can extend beyond test performance; stereotype threat can also affect students’ sense of belonging to the stereotyped domain, making them feel out of place in certain classes or majors (e.g., Good et al., 2012).

Stereotype threat theory specifies that negative stereotypes may only be threatening to individuals with high domain identification–in other words, only individuals who care about the threatened domain should experience stereotype threat (Steele et al., 2002). If succeeding in language classes is not relevant to a man’s identity, he should not be susceptible to a stereotype threat. One meta-analysis suggested that stereotype threat effect sizes were much larger among individuals who identified strongly with the threatened domain (*d* = 0.68 vs. *d* = 0.29; Walton and Cohen, 2003, p. 463). Another meta-analysis did not find a difference between moderate domain identification and high domain identification overall, but found that moderate domain identification resulted in the largest effect sizes in studies of women in mathematics specifically (Nguyen and Ryan, 2008, p. 1324).

There has also been debate and conflicting meta-analytic results regarding the use of subtle stereotype threat messages (e.g., describing the test as diagnostic of intelligence or priming the stigmatized identity) versus explicit stereotype threat messages (i.e., stating the expectation of a group-based performance difference). Some researchers have argued that more explicit threats (in contrast to subtle threats) might trigger stereotype reactance and improved performance rather than performance decrements (Kray et al., 2001), but no meta-analyses have reported evidence of such an effect. Nguyen and Ryan (2008) found that subtle stereotype threats yielded the largest effect sizes among women, but explicit threats had a greater effect on racial minority individuals. The former finding conflicts with two other meta-analyses that found that explicit threats produced larger effect sizes than subtle ones (Walton and Cohen, 2003, p. 463; Shewach et al., 2019) and one that yielded no moderation by type of threat (Flore and Wicherts, 2015).

### Gendered Stereotype Threats on Men

There is evidence suggesting that men may experience stereotype threat in language domains under certain circumstances. Keller (2007b) found that stereotype-threatened men performed poorly on verbal tasks under conditions of combined explicit stereotype threat and prevention focus, but not when unthreatened or manipulated to have a promotion focus. An experiment by Pansu et al. (2016) showed that boys who identified strongly with reading performed more poorly at a reading task after a subtle stereotype threat when controlling for reading ability. Van Loo et al. (2013) found that men primed with competition (as a subtle stereotype threat induction) performed more poorly on a verbal test than men in the control condition, whereas women were unaffected. Similarly, Hirnstein et al. (2014) found that subtle stereotype threat instructions decreased men’s performance on a verbal fluency task (which involved listing words beginning with certain letters and creating four-word sentences using words beginning with specific letters).

A few studies, however, have found opposite effects: Hirnstein et al. (2012) found that men’s performance on the same verbal fluency tasks as Hirnstein et al. (2014) was improved under a stereotype threat manipulation compared to a control condition. Hausmann (2014) also found that men performed better on one of these same verbal fluency tasks when gender stereotypes about such tasks were activated than when they were not. Still other studies have found null effects of stereotype threat on boys’ reading comprehension scores (Eckert and Imhof, 2013). In sum, results of how stereotypes affect men’s performance on language-related tasks have not been consistent, with some evidence pointing to stereotype threat, some evidence showing stereotype reactance, and some evidence suggesting no stereotype threat or reactance. We did not notice any clear systematic differences between the dependent variables of studies finding stereotype threat effects and those not finding them, and in fact, some studies have found opposite results with identical language tasks as dependent variables (Hirnstein et al., 2012, 2014). Furthermore, some results supporting a stereotype threat effect have been found only under specific conditions, and all have used native language tasks such as sentence construction, reading, or GRE-verbal type tests (Keller, 2007b; Van Loo et al., 2013; Pansu et al., 2016).

Missing from the research on men’s stereotype-threat experiences in language domains is the potential effect of stereotype threat on social–psychological outcomes such as sense of belonging. If stereotype threat does indeed affect men in language, it may lead individuals to disidentify with the threatened domain—in this case, it could lead male students to have a weaker language self-concept (Aronson et al., 1999), which could lead to anxiety and disinterest in language-related study. It might also lead men to devalue the threatened domain, adopting a more negative attitude about the value of language learning. This possibility has many potential implications for men’s educational choices, which shape men’s career opportunities in communicative fields. These stereotypes may also have implications for male immigrants’ intercultural contact experiences, including feelings of language anxiety and confidence or worry about facing rejection because of their language skills. Although it has been found that immigrants can experience language stereotype threat effects based on their linguistic background (Sander et al., 2018), gendered stereotype threats for this group have not yet been investigated to our knowledge.

If men experience stereotype threat in language and foreign language–related domains, interventions from the literature on women in STEM may be useful for reducing the effects; similar interventions may eliminate stereotype threat effects in men. One factor that has been effective in eliminating stereotype threat is mindset, or the individual’s beliefs about the nature of intelligence in the threatened domain (Good et al., 2003). In other words, individuals are more likely to experience stereotype threat when they endorse fixed mindsets, or beliefs that people have a certain capacity for learning something and that this capacity is genetically determined and cannot be altered or improved. On the other hand, individuals may not be susceptible to stereotype threat if they endorse growth mindsets, or beliefs that hard work and effort can allow one to improve one’s capacities (Good et al., 2003; Froehlich et al., 2016). Interventions to promote growth mindsets have been effective in eliminating stereotype threat effects (Aronson et al., 2002; Good et al., 2003; however, see Sisk et al., 2018 for a meta-analysis on growth mindset interventions that presents mixed results), which suggests that if men experience stereotype threat on verbal or foreign language tasks, these effects could be reversible through changing their mindsets.

## The Present Research

We conducted four stereotype threat experiments using different populations of university students, different manipulations for the comparison condition, and different dependent variables. These variables included foreign language learning aptitude measures and verbal tests in English to assess language test performance, measures of sense of belonging, and various measures of social psychological outcomes including devaluation of foreign language learning (measured as language attitudes), task motivation toward the language tests, and interest in language majors. Across all four studies, primary analyses were conducted using traditional null-hypothesis significance testing methods, but in order to quantify the relative likelihood of the null and alternative hypotheses, we followed these tests with Bayesian analysis of variance (ANOVA) using JASP software with a default prior of 0.50 (version 0.9.2; JASP Team, 2018).

## Study 1: Stereotype Threat and Language

The first study aimed to examine whether stereotype threat influences men’s performance on language tests and psychological outcomes related to language learning. We compared the effect of explicit stereotype threats with explicit stereotype negation. We also compared effects on men and women.

We chose to use explicit threat and explicit threat negation in Study 1. Stereotype negation conditions, which explicitly state that the stereotype is untrue or that group differences are not expected, rather than control conditions (in which no stereotype-relevant information would be mentioned), have been used to account for the possibility that individuals might be under a chronic stereotype threat—in other words, if stereotype threat is the default experience of men taking language tests, it might still operate in a control condition. Using explicit manipulations in both conditions was also intended to ensure that stereotypes other than the stereotype of interest would not become salient. In this study, we were interested in stereotypes about men and language, but we also wanted to ensure that the testing situation did not activate alternate stereotypes, such as stereotypes of general intelligence that might favor men. Although the stereotypes associating languages with women have been robust in previous studies (e.g., Plante et al., 2009), including recent studies of Canadian university students (Chaffee et al., 2020), stereotypes about men and language may be less culturally salient than stereotypes about women and STEM because the former is less often discussed than the latter.

### Hypotheses

Hypothesis 1-1: We expect that stereotype-threatened men will score more poorly on both the verbal test and the language aptitude test after stereotype threat compared to men in the threat-negated condition and compared to women in both conditions.
Hypothesis 1-2: We expect men in the threat condition to report a weaker sense of belonging to language domains than all other groups.
Hypothesis 1-3: We expect men in the threat condition to devalue language learning, reporting more negative language attitudes than all other groups. These men might also report lower task motivation compared to other groups, including putting less effort into the tasks and feeling more tense and less competent during the tests.

## Materials and Methods

### Participants

A total of 209 university students recruited from introductory psychology courses completed the study. Only participants who completed a pre-testing questionnaire and indicated moderate to strong identification (3 or higher on a 5-point scale) with language learning were invited to participate (because meta-analyses indicate that stereotype threat effect sizes are larger among students with moderate or high domain identification, e.g., Walton and Cohen, 2003). Fourteen participants who answered an attention check question incorrectly were excluded from analyses. Two female and three male participants in the threat condition were excluded because they correctly guessed the study hypothesis on the suspicion check. The final sample of 189 university students (95 female and 94 male) ranged from 17 to 37 years old (mean = 19.34 years, SD = 2.85 years). All participants were native speakers of English. The participants were randomly assigned to either a stereotype-threatened (47 women and 45 men) or threat-negated (48 women and 49 men) condition.

### Procedure

Across all four studies, the participants were tested in groups of up to 12. Each session was conducted by a female experimenter in a computer laboratory.

Participants were given a different set of study instructions depending on the experimental condition. Participants in the threat condition were told that gender differences in language aptitude exist and were relevant to the tests they would be taking, and participants in the threat-negated condition were told that gender differences in language ability did not exist for the tests they would complete. This manipulation was delivered both verbally by the experimenter and in writing on the questionnaire. Participants in the threat condition were asked to indicate their gender at the beginning of the questionnaire, whereas those in the threat-negated condition were asked at the end.

Following the manipulation, participants completed two language tests and a questionnaire. The order of questionnaire scales and items was randomized.

### Materials

#### Stereotype Threat Manipulation

The threat and threat-negated scripts (see Supplementary Material for full scripts for all studies) were adapted from Aronson et al. (1999) and reworded to refer to men and language, as well as to suit the Canadian context. One sentence was added to the end of each script specifying either that gender differences were expected (threat condition) or not expected (threat-negated condition) on the experimental tasks.

#### Verbal Test

Participants answered 11 multiple-choice questions from a practice SAT test (The College Board, 2014). Questions were chosen based on difficulty level (questions with the three highest difficulty levels out of five were selected) assigned by The College Board and pilot tested by the authors. Questions included in the present study were correctly answered by 52% or fewer of the (*N* = 87) pilot testers. Students had 10 min to complete the questions.

#### Language Aptitude Test

Participants also completed a computerized language aptitude test involving learning novel vocabulary (LLAMA B; Meara, 2005). The LLAMA tests are designed to measure cognitive capacities underlying the ability to learn new languages and are widely used by language researchers (e.g., Granena and Long, 2013; Rogers et al., 2017). Participants were given 90 seconds to learn nouns in a made-up language and then tested on their ability to recall the words. Scores on these tasks reflect a percentage score from 0 to 100%.

#### Attention Check

After the language tasks, participants were asked whether, according to the study description, the language tasks they completed tend to show gender differences favoring females, gender differences favoring males, or no gender differences.

#### Task Motivation and Effort

The Intrinsic Motivation Inventory (IMI) from Ryan (1982) was used to assess participants’ motivation toward the two language measures. The inventory consisted of 23 questions rated on a 7-point Likert scale that ranged from (1) “not at all true” to (7) “very true.” Seven items were used to assess intrinsic motivation (α = 0.89; “I enjoyed doing the language aptitude tasks very much”), six to assess competence (α = 0.89; “I think I am pretty good at these language aptitude tasks”), five to assess effort (α = 0.85; “I put a lot of effort into this”), and five to assess feelings of pressure during the task (α = 0.88; “I felt very tense while doing the language aptitude tasks”).

#### Sense of Belonging

The sense of belonging scale consisted of four questions adapted from the membership subscale developed by Good et al. (2012). Each question was assessed using an 8-point Likert scale of strongly disagree (1) to strongly agree (8; “I feel that I belong to the language community”; α = 0.93).

#### Language Learning Attitudes

The language learning attitude scale consisted of 10 questions adapted from Gardner et al. (1997). Each question was assessed using a 7-point Likert scale of strongly disagree (1) to strongly agree (7; “I would really like to learn many foreign languages”; α = 0.75).

#### Manipulation Check

Participants rated 11 questions on a 7-point scale ranging from “males are much better” (1) to “females are much better” (7). Participants rated their impressions of language-learning ability in the native language (five questions, α = 0.71) and in foreign languages (five questions, α = 0.73) in terms of the four basic skills (reading, writing, speaking, and listening comprehension), as well as in general. Participants also rated math ability on the same scale.

#### Suspicion Check

Participants reported what factors they felt influenced their performance on the language aptitude tasks and guessed the study hypothesis using two open-ended questions. Participants also answered a multiple-choice question to select at what point in the study they thought that their hypothesis was likely.

### Results and Discussion

#### *Post hoc* Power Analysis

Sensitivity analysis computed in G^∗^Power 3.0.10 (Faul et al., 2007) revealed that the study had 80% power to detect an effect size of *f* ≥ 0.24, suggesting that the present study had ample power to detect the effect size reported in Walton and Cohen’s (2003) meta-analysis (*f* = 0.34) for students who identified with the stereotype-threatened domain, as well the as the effect size (*f* = 0.26) reported in Nguyen and Ryan’s (2008) meta-analysis for moderately identified women in math. This study also used tests that were chosen and pilot tested for difficulty, which should yield larger effect sizes than other types of tasks (Nguyen and Ryan, 2008).

#### Manipulation Check

We found significant main effects of stereotype threat on beliefs in gender differences in both foreign language skills, *F*(1,180) = 35.17, *p* < 0.001, η^2^ = 0.16, Bayes factor 01 (BF_01_) < 0.01 (mean = 4.40, SD = 0.59 in the threat condition and mean = 4.07, SD = 0.40 in the non-threat condition for men; mean = 4.61, SD = 0.71 threat condition vs. mean = 4.05, SD = 0.23 non-threat for women), and native language skills, *F*(1,180) = 21.72, *p* < 0.001, η^2^ = 0.11, BF_01_ < 0.01 (mean = 4.53, SD = 0.60 threat vs. mean = 4.27, SD = 0.44 non-threat for men; mean = 4.71, SD = 0.62 threat condition vs. mean = 4.26, SD = 0.38 non-threat for women), such that participants in the threat condition believed in a stronger female advantage in both skills. This was consistent with the manipulation, which stated in the threat condition that female students outperform male students. Main effects of gender and interaction effects between gender and conditions were non-significant in both cases.

#### Major Analyses

**FIGURE 1:**
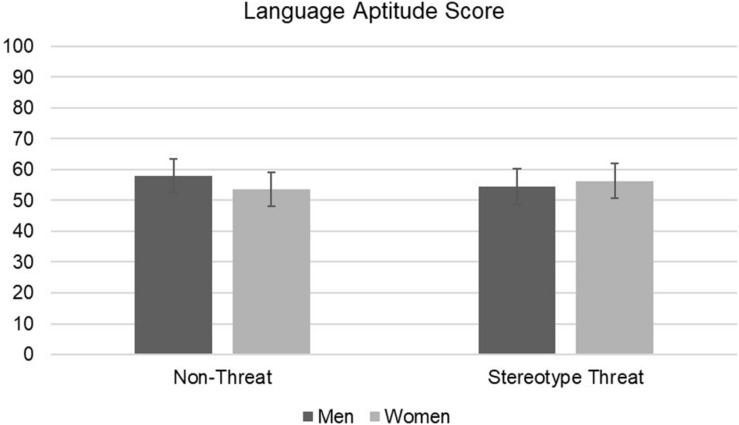
2 × 2 ANOVA results for language aptitude by gender and condition with 95% confidence interval bars.

**FIGURE 2:**
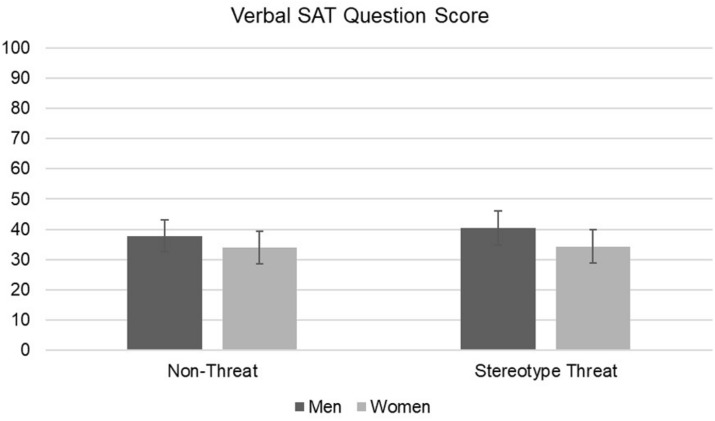
2 × 2 ANOVA results for verbal test by gender and condition with 95% confidence interval bars.

The results of 2 (gender) × 2 (condition) ANOVAs on different dependent measures are presented in Table 1. We found no significant main effects of gender or experimental condition and no significant interactions on language aptitude and SAT verbal test questions (Figures 1, 2). Similarly, we found no main effects of experimental condition, gender, or interaction effects for belongingness, language attitudes, or any IMI dimension (Table 1). We report BF_01_, which indicates the ratio of likelihood that the observed results would be obtained under a true null hypothesis compared to a true alternative hypothesis. Thus, a value of BF_01_ = 10.00 indicates that the null hypothesis is 10 times more likely than the alternative, and a value of BF_01_ = 0.10 would indicate that the alternative hypothesis is 10 times more probable than the null hypothesis.

**TABLE 1:** Results of 2 (men vs. women) × 2 (threat condition vs. threat-negated condition) ANOVA for Study 1.

		**Means (SD) Threat**		**Means (SD) Negated Threat**						
		**Men**	**Women**	**Men**	**Women**	***F***	**df**	***p***	**η^2^**	***BF*_01_**
Language aptitude		54.42 (20.15)	56.33 (18.26)	57.98 (19.66)	53.65 (19.09)					
	Gender					0.18	(1, 183)	0.672	<0.01	5.63
	Condition					0.02	(1, 183)	0.879	<0.01	6.18
	G × C					1.20	(1, 183)	0.275	<0.01	94.98
Verbal SAT questions		40.36 (19.21)	34.30 (16.74)	37.84 (21.22)	33.90 (17.99)					
	Gender					3.20	(1, 183)	0.075	<0.01	1.45
	Condition					0.27	(1, 183)	0.603	<0.01	5.53
	G × C					0.14	(1, 183)	0.705	<0.01	35.51
Belonging		4.40 (1.80)	4.88 (1.56)	4.95 (1.59)	5.27 (1.75)					
	Gender					2.56	(1, 183)	0.112	0.01	2.06
	Condition					3.53	(1, 183)	0.062	0.02	1.29
	G × C					0.30	(1, 183)	0.747	< (0.01	10.94
Language attitudes		5.48 (0.82)	5.61 (0.87)	5.68 (0.76)	5.81 (0.72)					
	Gender					1.29	(1, 184)	0.258	0.01	3.51
	Condition					2.91	(1, 184)	0.090	0.02	1.64
	G × C					0.00	(1, 184)	0.984	<0.01	25.01
Intrinsic motivation		4.04 (1.18)	3.97 (1.03)	4.11 (1.17)	3.96 (1.13)					
	Gender					0.45	(1, 184)	0.501	<0.01	5.01
	Condition					0.05	(1, 184)	0.847	<0.01	6.13
	G × C					0.07	(1, 184)	0.814	<0.01	32.12
Competence		3.59 (1.13)	3.20 (1.20)	3.76 (1.22)	3.31 (1.06)					
	Gender					6.05	(1, 184)	0.015	0.03	0.36
	Condition					0.63	(1, 184)	0.428	<0.01	4.60
	G × C					0.04	(1, 184)	0.837	<0.01	7.77
Effort		4.34 (1.27)	4.35 (1.25)	4.23 (1.35)	4.27 (1.04)					
	Gender					0.02	(1, 184)	0.887	<0.01	6.19
	Condition					0.25	(1, 184)	0.618	<0.01	5.55
	G × C					0.01	(1, 184)	0.913	<0.01	35.50
Pressure		3.47 (1.49)	3.59 (1.48)	3.17 (1.57)	3.38 (1.50)					
	Gender					2.32	(1, 184)	0.130	0.01	2.03
	Condition					0.83	(1, 184)	0.365	<0.01	4.21
	G × C					0.19	(1, 184)	0.661	<0.01	36.19

There were no differences in language aptitude between men and women. Counter to our expectations, stereotype threat did not negatively affect men’s performance on either test of language competence. Bayesian analysis indicated that the observed results for these interaction terms ranged from 7 to 94 times more likely under a true null hypothesis than a true alternative hypothesis. According to conventional interpretation, this constitutes strong to very strong evidence of the null hypothesis in the case of the two language tests (Lee and Wagenmakers, 2014).

## Further Studies

In the next two studies, we made several changes. The results of Study 1 suggested that participants were convinced by our stereotype threat manipulation, but the large BFs for the gender by condition interactions suggested that the manipulation nevertheless had no direct effect on participants’ language test performance (both SAT verbal questions and language aptitude). In Study 2, we examined stereotype threat in a specific group of language learners, English-as-a-second-language (ESL) students. In Study 3, we added a manipulation of mindsets, a known moderator of stereotype threat effects (e.g., Good et al., 2003). In the following studies (2–4), we replaced the threat-negated comparison condition with a non-threat control condition in which gender was not mentioned in the study instructions (see Supplementary Material for full scripts). We also chose not to select participants based on pre-tested language identification both to increase the potential generalizability of our findings and because meta-analytic results have been somewhat inconsistent with regard to what level of domain identification results in the strongest threat effects (Walton and Cohen, 2003; Nguyen and Ryan, 2008).

## Study 2: ESL Students’ Stereotype Threat on English

For migrants to Western Canada whose native language is not English, language is an important cultural tool and a foundational skill to daily life and functioning in Canadian society. Thus, studying ESL students’ stereotype threat on their English ability provides the opportunity to examine a group of men who (a) are all using the same target language of English and (b) should identify with the domain of English and feel it is self-relevant because of its central importance in their daily lives. Moreover, previous studies showed that men often feel less well adjusted to the new culture than women (Lee et al., 2009; Cole and McNulty, 2011) and also that students from immigrant backgrounds can experience linguistic stereotype threat based on this background (Sander et al., 2018), but our examination of gendered stereotype threat on this group is a novel contribution of this study. Given that language skill is one of the most powerful predictors of sociocultural adaptation (Noels et al., 1996; Wilson et al., 2013), the stereotype that men have comparatively poor language ability may have significant implications for the acculturation of male international students and immigrants. Specifically, stereotype-threatened men might feel less confident in using English and see their sense of belonging to the whole host culture negatively affected by stereotypes about men’s language ability.

### Hypotheses

Compared to ESL men who are not threatened, we expect that stereotype-threatened ESL men will

Hypothesis 2-1: Perform more poorly on the English verbal test and the language aptitude test.
Hypothesis 2-2: Experience greater anxiety about both using English and interacting with Anglo-Canadians, more avoidance tendencies around interacting with Anglo-Canadians, as well as greater sensitivity to the possibility of rejection because of their language skills. We also expect participants to report poorer acculturation after stereotype threat, and possibly lower self-esteem.

### Participants

Male students in introductory psychology classes who were born outside Canada and did not have English as a native language participated in the study. Nine students in the stereotype activated condition and 13 students in the non-threat (control) condition were excluded because they answered the attention check question incorrectly. Seven students, all in the stereotype activated condition, were excluded because they correctly identified the hypothesis of the study on the suspicion check questions. The final sample consisted of 105 male international and immigrant students. Participants’ native languages included Chinese languages (*n* = 45), south Asian languages (*n* = 14), European languages (*n* = 8), and several other languages represented by fewer than five speakers. Participants were randomly assigned to either threat (*n* = 53) or non-threat (*n* = 52) conditions. Participants’ ages ranged from 17 to 63 years (mean = 21.21, SD = 5.04).

### Materials

The same SAT verbal test questions, language aptitude test, belongingness measure, and task motivation measures were used as in Study 1.

#### English Anxiety and Confidence

These scales were adapted from Gardner (2010; anxiety α = 0.89, “I never feel quite sure of myself when I am speaking in English”; confidence α = 0.94, “I feel confident when I speak in English”) and were rated from 1 (“strongly disagree”) to 7 (“strongly agree”).

#### Language-Based Rejection Sensitivity

Participants’ expectations of being rejected because of their status as non-native English speakers (e.g., “I would expect that the receptionist might talk to me impatiently because I am not a native English speaker”; α = 0.86) and their feelings of anxiety about this possibility (e.g., “How concerned/anxious would you be that the receptionist might talk to you impatiently because you are not a native English speaker”; α = 0.93) were measured using a scale from Lou and Noels (2019). Participants rated each item from 1 (“very unlikely/very unconcerned”) to 6 (“very likely/very concerned”). For each item, expectation and anxiety were multiplied before the mean score was computed. The overall reliability was α = 0.86.

#### Intergroup Anxiety, Avoidance, and Hostility

A scale adapted from Plant and Devine (2018) was used to measure intergroup anxiety (“I would feel uncomfortable when interacting with an English-speaking white Canadian”; α = 0.88), intergroup avoidance (“If I had a choice, I would rather not interact with an English-speaking white Canadian”; α = 0.86), and intergroup hostility (“I would find interacting with an English-speaking white Canadian annoying”; α = 0.94). Participants rated each item from 1 (“strongly disagree”) to 7 (“strongly agree”).

#### Cross-Cultural Adjustment

Feelings of adjustment to Canada in various domains were measured using a scale adapted from Black and Stephens (1989). On a scale from 1 (not at all adjusted) to 7 (very well adjusted), participants rated how well they felt they had acculturated to 14 aspects of life in Canada such as “the academic requirements,” “interpersonal relationships,” and “customs and practices” (α = 0.93).

#### Self-Esteem

Self-esteem was measured using the Rosenberg self-esteem scale (10 items; “On the whole, I am satisfied with myself” α = 0.88). Participants rated each item from 1 (“strongly agree) to 4 (“strongly disagree”).

### Results and Discussion

Sensitivity analysis computed in G^∗^Power 3.0.10 revealed that the study had 80% power to detect an effect size of *f* ≥ 0.28.

There were no significant effects of stereotype threat. Threat did not affect performance on the language aptitude tasks or English verbal test, sense of belonging in language, rejection sensitivity, feelings of adjustment, English anxiety, or intergroup contact emotions or intentions (Table 2). Bayesian analyses revealed moderate support for the null hypothesis, ranging from almost three times more likely than the alternative hypothesis, to more than four and a half times more likely.

**TABLE 2:** Results of one-way analyses of variance by condition for Study 2.

	**Threat**		**Non-threat**						
	**Mean**	**SD**	**Mean**	**SD**	***F***	**df**	***p***	**η^2^**	***BF*_01_**
Language aptitude	36.33	18.67	38.73	15.93	0.48	(1, 99)	0.491	0.01	3.83
Verbal test questions	29.42	15.24	28.15	17.64	0.15	(1, 102)	0.697	<0.01	4.49
Belonging	5.08	1.55	4.97	1.54	0.02	(1, 105)	0.730	<0.01	4.60
Rejection sensitivity	8.12	5.45	8.36	5.32	0.05	(1, 102)	0.819	<0.01	4.78
Adjustment	5.21	1.02	5.30	1.06	0.19	(1, 89)	0.662	<0.01	4.16
English anxiety	3.56	0.99	3.43	1.04	0.36	(1, 77)	0.550	0.01	3.64
Intergroup anxiety	2.83	1.19	2.59	1.03	0.82	(1, 71)	0.368	0.01	2.89
Intergroup avoidance	2.66	1.22	2.47	1.09	0.46	(1, 71)	0.500	0.01	3.38
Intergroup hostility	2.23	1.16	2.01	1.07	0.70	(1, 70)	0.407	0.01	3.03

## Study 3: Stereotype Threat and Mindsets

In Study 3, we examined whether a mindset intervention would interact with stereotype threat on men’s language ability. Previous research has shown that mindsets moderate stereotype threat such that stereotype threat is experienced by individuals with fixed mindsets, but not by those with growth mindsets (Good et al., 2003). By manipulating mindset, we expected to find a stereotype threat effect among men primed with a fixed mindset. In other words, it was expected that threatened men would perform worse on the language tasks and express less belonging to language domains if they also had a fixed mindset of language intelligence. We expected that men primed with a growth mindset would be unaffected by stereotype threat and that these men would perform at least as well as men exposed to fixed mindset but not stereotype threat.

The language competence–dependent variables were also modified for Study 3. Because stereotype threat effects have been found to be larger on difficult tasks (Nguyen and Ryan, 2008), we hoped to find significant stereotype threat effects in this study by increasing the difficulty of the language tests, as well as increasing their subjective difficulty for the participants by increasing how much time pressure the participants were likely to experience. Specifically, the LLAMA language aptitude task was made more difficult by reducing the time for the learning phase, and an anagram-solving task involving 20 challenging anagrams replaced the 11 SAT questions used in Study 1.

### Hypotheses

Hypothesis 3-1: Participants primed with a growth mindset are not expected to experience negative effects from stereotype threat. Men in the stereotype-threatened condition who are also primed with a fixed mindset are expected to perform more poorly on the language tests than other groups.
Hypothesis 3-2: Men in the stereotype-threatened condition who are also primed with a fixed mindset are expected to feel less belonging to language domains than men primed with a growth mindset or men in the non-threat condition. These men may also report less task motivation than other groups.

### Participants

A total of 167 English-speaking male students participated in the present study in exchange for partial course credit. Although participants reported their domain identification with language in a pre-test session, we did not select participants based on this domain identification in Study 3. Participants were randomly assigned to the four experimental conditions. Participants who failed attention checks or reported suspicion of the study hypotheses were excluded from analysis: six men reported suspicions, 19 failed the attention check for the threat manipulation, and four participants failed the attention check for the mindset article. The final sample consisted of 138 male students aged 18 to 30 years (mean = 19.63, SD = 1.93).

### Procedure

Procedures for the session and the stereotype threat manipulation were the same as for Study 1, except that a control condition in which no reference was made to any gender differences in language learning was used instead of a threat-negated condition.

Also added for Study 3 was a second independent variable manipulation. After the study instructions, participants were told to read an article, ostensibly for a reading comprehension test, but actually intended to manipulate participants’ belief in either an incremental or entity theory of language intelligence. The participants were randomly assigned to read one of the two articles developed by Lou and Noels (2016). The growth mindset article stated that language ability is largely determined by environmental factors and can be improved through effort, whereas the fixed mindset article stated that language ability is unchangeable and determined by genetics. The threat-activated group consisted of 60 individuals, 28 in the entity condition and 32 in incremental condition. The non-threat (control) group consisted of 87 individuals, 45 in the entity condition and 42 in the incremental condition.

Following the two manipulations, participants completed two language tests (anagrams and a vocabulary learning activity) and a questionnaire.

### Materials

The language aptitude task (LLAMA B), task motivation, and language attitudes measures from the previous studies were used.

#### Incremental and Entity Theory of Language Intelligence Manipulation

The participants read one of the two articles from Lou and Noels (2016) described above. The full manipulation articles are available at https://osf.io/2xu36/ beginning on page 9 of the supplement.

#### Anagrams Completion Task

The participants were told the definition of an anagram and given 5 min to complete as many anagrams as possible from a list of 20.

#### Task Motivation and Effort

The IMI from Ryan (1982) was again used, and Cronbach α’s ranged from 0.88 to 0.91.

#### Foreign Languages

The participants were asked to indicate whether they were currently studying any second or foreign languages, whether they planned on studying any second or foreign languages in the future, and whether they spoke any other languages in addition to their native language(s). They were also asked to list the foreign language(s) they had studied in the past.

#### Sense of Belonging

The full 30-item sense of belonging scale (adapted from Good et al., 2012) was used to measure the participants’ feelings in language settings. The participants rated the items on a scale from (1) “strongly disagree” to (8) “strongly agree.” The scale included five subscales: four questions were used to assess participants’ sense of membership in the language setting, as in Study 1 (“I feel that I belong to the language community”; α = 0.88), 10 to assess their sense of acceptance (“I feel like an outsider”; α = 0.76), eight to assess their affect in the language setting (“I feel at ease”; α = 0.91), four to assess a desire to fade away (“I wish I could fade into the background and not be noticed”; α = 0.79), and four to assess trust (“I trust the test materials to be unbiased”; α = 0.67). Because including the item (“I have trust that I do not have to constantly prove myself”) in the trust subscale lowered the Cronbach’s α value to 0.57, it was excluded from analysis.

#### Manipulation, Attention, and Suspicion Checks

The same questions as Study 1 were used to assess participants’ attention to the threat manipulation and their suspicion of the study hypotheses. To bolster the cover story about the mindset articles, the attention and comprehension check for this manipulation was embedded in a “reading comprehension and retention task” consisting of five filler questions and two attention check questions: “According to the article, what are the roles of genetic and environmental factors in respect to language intelligence?” and “Which results did Knowles find in the study done with twins in terms of their language intelligence?” These two questions were used to determine whether participants read and understood the article. Participants who chose an answer that did not correspond to the article they were assigned were removed from analyses.

#### Language Mindsets

The Language Mindsets Inventory (Lou and Noels, 2017) was used as a check for the mindset manipulation. Each item was assessed using a 6-point Likert scale ranging from strongly disagree (1) to strongly agree (6). Fixed beliefs mindsets (nine items; “People have a certain amount of language intelligence, and people can’t really do much to change it”) and growth mindsets (nine items; “No matter who the person is, people can significantly change their language intelligence level”) were collapsed into a single index for which a high score indicated a strongly fixed mindset and a weak growth mindset, whereas a low score reflected a strong growth mindset and weak fixed beliefs (α = 0.92).

### Results and Discussion

Sensitivity analysis computed in G^∗^Power 3.0.10 revealed that the study had 80% power to detect an effect size of *f* ≥ 0.29. There was a main effect of article on mindsets such that participants who read the fixed article reported language mindsets that were more fixed and less incremental (mean = 3.42, SD = 0.80) than participants who read the growth article (mean = 2.63, SD = 0.74) *F*(1,136) = 36.14, *p* < 0.001, suggesting that the mindset manipulation was effective.

Results of 2 (mindset conditions) × 2 (threat conditions) ANOVAs revealed no significant main effects of mindset condition, threat condition, or their interactions on any of the dependent variables; stereotype threat, mindset manipulation, and their interaction did not affect language aptitude, anagram solving performance, attitudes toward foreign languages, or any subscale of sense of belonging (Table 3). Overall, the results suggested no effects of the stereotype threat on men.

**TABLE 3:** Results of 2 × 2 analyses of variance by stereotype threat (threat vs. non-threat) and mindset article (growth vs. fixed) conditions for Study 3.

		**Means (SD) Threat**		**Means (SD) Non-threat**						
		**Growth**	**Fixed**	**Growth**	**Fixed**	***F***	**df**	***p***	**η^2^**	**BF**_01_
Language aptitude		42.64 (15.24)	44.81 (14.53)	45.78 (19.18)	44.58 (15.23)					
	Article					0.03	(1, 130)	0.866	<0.01	5.23
	Condition					0.35	(1, 130)	0.557	<0.01	4.52
	A × C					0.26	(1, 130)	0.611	<0.01	84.15
Anagrams		64.20 (22.27)	69.95 (21.22)	72.80 (22.45)	54.11 (23.33)					
	Article					0.14	(1, 134)	0.709	<0.01	5.26
	Condition					0.12	(1, 134)	0.726	<0.01	2.77
	A × C					3.36	(1, 134)	0.069	0.03	17.15
Language attitudes		5.19 (1.14)	5.41 (0.95)	5.62 (0.95)	5.19 (0.99)					
	Article					0.33	(1, 138)	0.567	<0.01	4.08
	Condition					0.35	(1, 138)	0.558	<0.01	4.55
	A × C					3.44	(1, 138)	0.066	0.03	17.48
Membership		3.91 (1.60)	4.01 (1.31)	4.33 (1.66)	4.28 (1.57)					
	Article					0.01	(1, 138)	0.930	<0.01	5.45
	Condition					1.66	(1, 138)	0.200	0.01	2.49
	A × C					0.07	(1, 138)	0.798	<0.01	52.74
Acceptance		5.04 (1.14)	4.80 (1.04)	5.14 (1.14)	4.99 (1.08)					
	Article					1.03	(1, 138)	0.312	0.01	4.21
	Condition					0.62	(1, 138)	0.432	0.01	4.68
	A × C					0.06	(1, 138)	0.816	<0.01	73.10
Affect		4.74 (1.27)	4.67 (1.81)	4.90 (1.37)	4.77 (1.28)					
	Article					0.21	(1, 138)	0.648	<0.01	5.22
	Condition					0.35	(1, 138)	0.554	<0.01	4.78
	A × C					0.02	(1, 138)	0.898	<0.01	103.16
Invisibility		3.91 (1.60)	3.71 (1.16)	3.29 (1.41)	3.98 (1.59)					
	Article					0.93	(1, 138)	0.337	0.01	2.91
	Condition					0.45	(1, 138)	0.505	<0.01	4.38
	A × C					3.11	(1, 138)	0.080	0.02	13.39
Trust		5.27 (1.18)	5.05 (1.09)	5.06 (1.34)	5.08 (1.16)					
	Article					0.21	(1, 138)	0.650	<0.01	4.96
	Condition					0.19	(1, 138)	0.664	<0.01	4.57
	A × C					0.34	(1, 138)	0.564	<0.01	86.26

## Study 4: Stereotype Threat, Aptitude and Interest

In Study 4, we measured four different aspects of language aptitude rather than only the ability to learn vocabulary. To explore whether stereotype threat might affect men’s educational preferences and choices related to language, we also added a measure of participants’ interest in various language-related and non–language-related major subjects. Because almost all students at Canadian universities have experience with second language learning before university, we measured participants’ willingness to communicate in whichever language they had studied longest in the past, as it is likely that men whose competence in language is threatened might feel hesitant to speak other languages as a result. We also reincluded the manipulation check from Study 1 to confirm that men were convinced by the threat information.

### Hypotheses

Hypothesis 4-1: Stereotype threat will result in lower scores on a language aptitude test. To determine whether stereotype threat effect will appear only on certain facets of language aptitude, we included all four subtests of the LLAMA as our language aptitude outcomes.
Hypothesis 4-2: Men’s sense of belonging to language domains is expected to be negatively impacted by stereotype threat.
Hypothesis 4-3: Stereotype threat will depress men’s interests in foreign language subjects and careers, but not STEM. Stereotype threat will lead men to be less willing to communicate in a previously studied foreign language.

### Methods

#### Participants

A sample of 139 male native English speakers enrolled in introductory psychology courses at a Canadian university participated in the study in exchange for partial course credit. Students who answered the attention check question incorrectly (nine students in the stereotype activated condition and 13 in the control condition) were excluded from analyses. Seven students, all in the stereotype activated condition, were excluded because they correctly identified the hypothesis of the study on the suspicion check questions. As a result, 110 participants remained in the sample, with 53 randomly assigned to the stereotype activated condition and 57 to the non-threat (control) condition. Participant ages ranged from 17 to 37 years (mean = 19.16 years), SD = 2.74 years), and 16.4% of the participants were studying a second or foreign language at the time of the study. Just over half (51.8%) of participants spoke another language in addition to their native language.

### Materials

Materials are the same as Study 3, with exceptions as follows:

#### Language Aptitude Tasks

In addition to LLAMA B task, LLAMA F assessed grammatical inferencing, LLAMA E assessed the relationship between sounds and a writing system, and LLAMA D assessed participants’ ability to recognize sound patterns in spoken language.

#### Willingness to Communicate

McCroskey’s (1992) scale was used to assess participants’ willingness to communicate in a language other than English. The scale asks participants to indicate what percentage of the time they would communicate in a foreign or second language in 20 given situations (e.g., “Talk with a friend while standing in line”; α = 0.97).

#### Belief in Gender Differences

The same 11 questions as in Study 1 were used to assess participants’ beliefs in gender differences in language ability. Participants rated the four language skills and general language ability along a 5-point scale ranging from “males are much better” (1) to “females are much better” (5) in both native and foreign language. In this study, however, native language proficiency showed poor reliability (α = 0.56), although foreign language skill remained reliable (α = 0.74). The two mean scores were analyzed as a manipulation check despite the low reliability for native language.

#### Subject and Career Interest

Participants were asked to indicate how interested they were in studying 13 different subjects. These included three foreign languages (French, Spanish, and German), three other language related subjects (East Asian Studies, English, and Linguistics), five STEM majors, Nursing, and Psychology. Next, participants were asked how interested they were in 16 different careers related to either language (e.g., Translation, Airline services) or STEM (e.g., Scientist, Engineer) or known to be highly gendered. All questions were on a 5-point scale ranging from 1 (“not at all interested”) to 5 (“very interested”).

#### Intention to Study Foreign Languages

Participants were asked if they were studying any second or foreign language at the time of the study and also if they had done so in the past. Additionally, participants were asked if they plan to study a second or foreign language in the future.

### Results and Discussion

#### Preliminary Analyses

Sensitivity analysis computed in G^∗^Power 3.0.10 revealed that this study had 80% power to detect an effect size of *f* ≥ 0.27. The results of a one-way ANOVA showed that the stereotype threat manipulation increased participants’ belief in a gender difference favoring women in both foreign language ability, *F*(1,94) = 4.81, *p* = 0.031, η^2^ = 0.05, *BF*_10_ = 0.57 (mean = 3.37, SD = 0.44 in the threat condition and mean = 3.21, SD = 0.30 in the non-threat condition), and native language ability, *F*(1,94) = 4.51, *p* = 0.036, η^2^ = 0.05, *BF*_10_ = 0.65 (mean = 3.32, SD = 0.53 in the threat condition and mean = 3.12, SD = 0.32 in the non-threat condition), although the BFs approached 1, indicating that the null hypothesis was almost equally likely.

#### Major Analyses

Main effects of stereotype threat on all facets of language aptitude, as well as a sense of belonging, language attitudes, language competence, foreign language career interest, and willingness to communicate in a foreign language, were non-significant (Table 4). After applying Bonferroni correction for multiple comparisons, interests in all language majors were also non-significant. Bayesian analyses showed slightly to moderately more support for the null hypothesis than the alternative hypothesis for main effects of stereotype threat on all dependent variables except interest in Spanish and French majors; for both Romance languages, the probabilities of the null and alternative hypotheses were similar (Table 4)^1^.

**TABLE 4:** Results of one-way ANOVA by threat condition for Study 4.

	**Threat**		**Non-threat**						
	**Mean**	**SD**	**Mean**	**SD**	***F***	**df**	***p***	**η^2^**	***BF*_01_**
**Language aptitude**									
LLAMA B	55.29	23.94	50.45	23.32	1.10	(1, 104)	0.297	0.01	2.97
LLAMA F	54.46	25.49	55.09	26.10	0.02	(1, 107)	0.899	<0.01	4.87
LLAMA E	73.51	28.15	75.44	26.66	0.13	(1, 107)	0.715	<0.01	4.61
LLAMA D	30.61	15.95	30.18	14.64	0.02	(1, 107)	0.883	<0.01	4.85
Belonging	5.18	1.44	4.86	1.62	1.13	(1, 105)	0.290	0.01	2.93
Language attitude	5.55	0.70	5.69	0.74	0.99	(1, 106)	0.323	0.01	3.14
Language competence	4.33	0.81	4.38	0.92	0.08	(1, 109)	0.782	<0.01	4.78
**Interest in language majors**									
French	2.97	1.16	3.38	1.21	2.56	(1, 88)	0.114	0.03	1.47
Spanish	2.87	1.32	3.46	1.20	4.82	(1, 87)	0.031	0.05	0.55
German	2.84	1.35	3.00	1.31	0.30	(1, 86)	0.583	<0.01	3.88
English	2.88	1.45	3.06	1.39	0.38	(1, 88)	0.540	<0.01	3.80
Foreign language career interest	1.56	0.91	1.64	0.99	0.16	(1, 83)	0.693	<0.01	3.33
Interest in STEM majors	3.06	0.98	2.92	0.97	0.44	(1, 90)	0.510	<0.01	3.72
STEM career interest	1.90	1.18	1.82	0.98	0.10	(1, 83)	0.750	<0.01	4.15
Willingness to communicate in a foreign language	31.47	24.58	36.15	24.52	0.94	(1, 102)	0.335	0.01	3.17

## Summary of Four Studies

### Mini–Meta-Analyses

**FIGURE 3:**
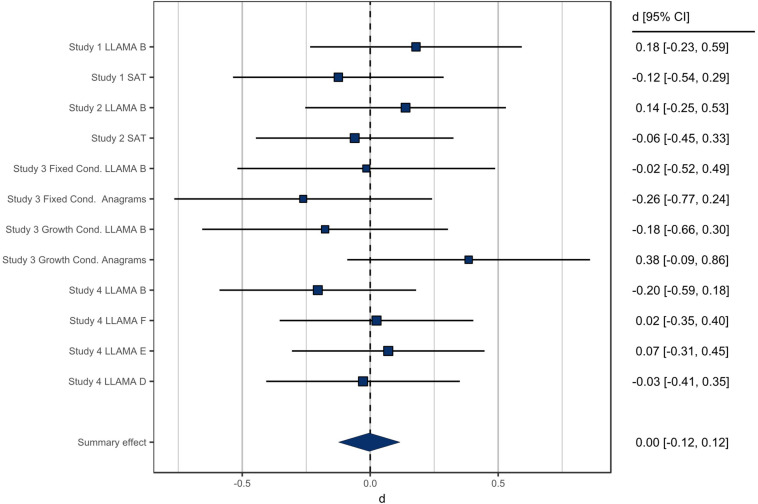
Mini meta-analysis of stereotype threat on men’s language task performance. Positive effect sizes are effect sizes in the expected direction (i.e., poorer performance among stereotype-threatened men), whereas negative effect sizes represent stereotype-threatened men outperforming unthreatened men.

**FIGURE 4:**
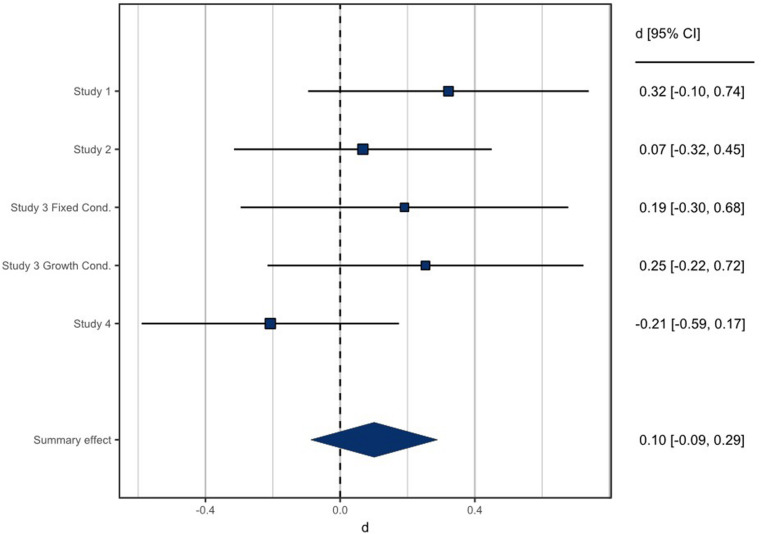
Mini meta-analysis of stereotype threat on men’s sense of membership to language communities. Positive effect sizes are effect sizes in the expected direction (i.e., less sense of belonging among stereotype-threatened men), whereas negative effect sizes represent stereotype-threatened men reporting stronger membership than unthreatened men.

In order to summarize effect sizes for stereotype threat effects on men across these four studies, we conducted fixed effect mini meta-analyses following the recommendations of Goh et al. (2016). The dependent variables that were consistent across all studies were language test performance and sense of membership to language domains. These sets of variables were each meta-analyzed among only men, with the two article conditions from Study 3 separated and the women in Study 1 omitted. As shown in Figure 3, the overall effect size of stereotype threat on men’s language task performance was less than one-hundredth of one standard deviation (*d* < 0.01, *p* = 0.951, 95% confidence interval = [−0.124, 0.116], *k* = 12). The effect size for sense of membership also failed to differ significantly from 0 (Figure 4; *d* = 0.10, *p* = 0.293, 95% confidence interval = [−0.087, 0.289], *k* = 5).

## General Discussion

Across four studies, we found no evidence that an explicit stereotype threat directly impairs men’s performance on language tests or suppresses men’s belonging in language domains. In Study 1, we also found no significant gender differences on our language tests–in fact, men scored slightly (but not significantly) higher than women on the SAT questions, suggesting that men were not underperforming on our language performance tasks. We also found no stereotype threat effects on social psychological outcomes such as attitudes toward the value of learning foreign languages or migrant ESL students’ adjustment to Canada. Despite previous research suggesting that Canadian university students believe that women and girls have stronger verbal and foreign language ability than men and are aware of cultural stereotypes that language is “for girls” (Chaffee et al., 2020), we found little evidence of stereotype threat on male students, and Bayesian analyses indicate support for the null hypothesis.

Study 4 showed inconclusive results with regard to whether stereotype threat might suppress men’s interest in studying certain foreign languages; the probability of the alternative hypothesis was similar to the likelihood of a true null hypothesis for interest in French and Spanish. It is unclear why a competence threat would affect interest in certain languages without affecting outcomes typically associated with stereotype threat such as test performance, sense of belonging, or attitudes about language learning. In light of the possibility that different languages might be differentially gendered, future studies should also examine a greater number of languages.

### Limitations and Future Directions

The present studies showed consistent results, but some features may limit their generalizability. First, the comparison conditions varied across studies, but all studies used an explicit stereotype threat induction procedure. It is possible that a subtle stereotype threat message might elicit stereotype threat effects for men in language domains. Second, all participants were recruited from introductory psychology classes. Participants in Study 1 were selected for moderate to high domain identification with language, and participants in Study 2 were students undertaking postsecondary education in a non-native language and were therefore presumed to be a population for whom language would be particularly self-relevant, but psychology students are nevertheless a population for whom language skills might not be of central importance. Because introductory psychology is an extremely popular course taken by a wide cross-section of university students from all majors, this provides a relatively heterogeneous sample. It remains possible that men specifically in humanities majors might be susceptible to stereotype threat effects. It is also possible that stereotype threat effects might exist among younger boys in primary or secondary school, as we studied only young adult university students. A final possibility is that stereotype threat effects for men in language exist, but are extremely small in size and beyond our ability to detect in these studies.

Other research has demonstrated that boys might prefer STEM subjects and careers to language arts as the result of stereotypes that boys are comparatively stronger in STEM than in language-related skills, rather than as a result of stereotypes that compare them to girls (Plante et al., 2019) and that gendered stereotypes about language arts are associated with less interest in language-related fields and poorer language arts grades among boys (Plante et al., 2013). These findings might apply to foreign language contexts as well. Chaffee et al. (2020) have found that men’s traditional beliefs about masculine gender roles lead men to devalue foreign-language learning and distance themselves from interest in foreign languages following a threat to their masculine prototypicality. Stereotypes that FL is a feminine domain remain relevant not because reminders of competence stereotypes have a direct effect on men, but because men might devalue FL as a result of believing that feminine pursuits are not appropriate for men.

## Conclusion

Recently, researchers have raised questions around stereotype threat. Some have questioned whether stereotype threats lead to practically meaningful effect sizes (Shewach et al., 2019); others have been unable to replicate stereotype threat effects for women and girls in math (Ganley et al., 2013; Pennington et al., 2018; Flore et al., 2019), and still others are working on a meta-analysis examining whether the magnitude of stereotype threat effects is decreasing over time (Lewis and Michalak, 2019). Results of stereotype threat research on men in language in particular have been inconsistent, with significant findings reported for both impaired and improved performance after threat (Hirnstein et al., 2012; Pansu et al., 2016), as well as null results (Eckert and Imhof, 2013) and our present results showing evidence of no effect. Many researchers are concerned about publication bias, which leads to the systematic non-publication of non-significant results. This bias contributes to problems such as uncertainty about the replicability and true effect sizes of psychological phenomena (Ferguson and Heene, 2012). Shewach et al. (2019) found evidence that this publication bias is a problem for the stereotype threat literature specifically. Another benefit of studies that probe null findings is that they may help the research community to identify boundary conditions for psychological phenomena, revealing conditions under which stereotype threats tend not to occur and allowing future research to be directed toward other explanations. As a result, null results such as those found by the present series of studies make a valuable contribution to the literature.

Overall, Bayesian analyses of our four studies revealed moderate to strong evidence that stereotype threats to men’s perceived language competence did not affect men in terms of either their performance on language-related tests or their attitudes or feelings of belonging to language classes. Although our manipulations appeared to be effective in changing men’s beliefs about gender and language ability, this did not lead to any particular negative outcomes for threatened men. Although participants believed the threat information, one possibility is that they may not have felt threatened by it. This potential explanation is supported by the fact that participants in the threat condition did not feel greater pressure than those in the comparison condition in Study 1; nor did participants in Study 2 report greater English anxiety or intergroup anxiety after threat. The null results of stereotype threat in our studies held even under circumstances that ought to enhance stereotype threat effects, such as among men who identified with language domains (Study 1) or for whom language could be expected to be self-relevant and important (Study 2) and men primed with fixed mindsets (Study 3). The conclusion that explicit stereotype threats did not affect men’s outcomes is further supported by our mini meta-analyses, which show an aggregate effect indistinguishable from zero for the effect of stereotype threat on men’s performance and an extremely small effect that does not differ significantly from zero for stereotype threat effects on men’s sense of membership to language communities. We consistently found null effects across multiple populations, including English-speaking men and ESL students. Our null results were also consistent across multiple types of language tests and multiple social psychological outcomes. Overall, we found evidence that explicit stereotype threats have no effect on language outcomes for men enrolled in Canadian universities.

This evidence casts doubt on whether stereotype threat is a major factor explaining men’s underperformance on standardized tests of language or men’s underrepresentation in elective foreign language classes and majors. Given that stereotypes about men and language are strong and real-life differences in language course enrollment are large, it seems likely that other factors, such as beliefs about what interests, fields of study, and behaviors men believe are appropriate for them (e.g., traditional masculinity ideologies; see Chaffee et al., 2020), may have greater explanatory power.

## Data Availability Statement

The datasets generated for this study are available on request to the corresponding author.

## Ethics Statement

The studies involving human participants were reviewed and approved by the Research Ethics Office of the University of Alberta. The patients/participants provided their written informed consent to participate in this study.

## Author Contributions

KC and NL developed the study concept and study designs with input from KN. NL and KC analyzed and interpreted the data under the supervision of KN. KC drafted the manuscript with feedback and revision from NL and KN. All authors approved the final manuscript version.

## Conflict of Interest

The authors declare that the research was conducted in the absence of any commercial or financial relationships that could be construed as a potential conflict of interest.

## Footnotes

1 Paired-samples *t* tests revealed no significant differences between interest in foreign language and STEM majors *t*(88) = −1.19, *p* = 0.236 or careers *t*(82) = 1.73, *p* = 0.088.
