# Supplementary material for: Does Stereotype Threat Affect Men in Language Domains?
Source: Front Psychol. 2020 Jul 8;11:1302. doi: 10.3389/fpsyg.2020.01302 (PMC7360796; doi:10.3389/fpsyg.2020.01302)
Supplement: Supplementary file 1 [file Table_1.DOCX]

**Threat Condition (Studies 1-4)**

As you probably know, language skills are crucial to performance in many important subjects in university. Yet surprisingly little is known about the mental processes underlying language aptitude. This research is aimed at better understanding what makes some people better at languages than others.

As you also may know, at some top schools, female students outnumber the male students in language majors and majors with language as a prerequisite, and there seems to be a growing gap in academic performance between these groups. A good deal of research indicates that females consistently score higher than males on standardized tests of language ability. But thus far, there is not a good explanation for this. The research you are participating in is aimed at better understanding these differences. Your performance on the exam will be compared to other students from across Canada. One specific question is whether females are superior at all aspects of language aptitude or only certain types. This test measures your language aptitude, or how well you are able to learn languages.

The test you are about to take is one that has shown gender differences in the past.

**Negated Threat Condition (Study 1)**

As you probably know, language skills are crucial to performance in many important subjects in university. Yet surprisingly little is known about the mental processes underlying language aptitude. This research is aimed at better understanding what makes some people better at learning languages than others. Your performance on the exam will be compared to other students from across Canada. This test measures your language aptitude, or how well you are able to learn languages.

The test you are about to take is one that has not shown any gender differences in the past.

**Non-Threat Condition (Studies 2-4)**

As you probably know, language skills are crucial to performance in many important subjects in university. Yet surprisingly little is known about the mental processes underlying language aptitude. This research is aimed at better understanding what makes some people better at learning languages than others. Your performance on the exam will be compared to other students from across Canada. This test measures your language aptitude, or how well you are able to learn languages.
